# ID 322 - Análise de Resultados dos Relatórios de Avaliação Crítica dos Dossiês Submetidos à Agência Nacional de Saúde

**DOI:** 10.5327/2237-9622.2025.v34s1.322

**Published:** 2025-11-25

**Authors:** Letícia Brust, Andre Luis Ferreira de Azeredo da Silva, Maria Angélica Pires Ferreira

**Keywords:** Avaliação de Tecnologias em Saúde, saúde suplementar, avaliação econômica em saúde

## Abstract

**Introdução:**

O Núcleo de Avaliação de Tecnologias em Saúde do Hospital de Clínicas de Porto Alegre (Nats-HCPA) vem atuando como parceiro da Agência Nacional de Saúde Suplementar (ANS) para a avaliação crítica de solicitações de incorporação de tecnologias da saúde no rol de procedimentos por meio dos relatórios de avaliação crítica (RACs) desde 2022. A análise dos RACs pode ser utilizada para se obter um melhor entendimento acerca do processo de avaliação de tecnologias na saúde suplementar e para avaliar necessidades de melhorias.

O objetivo principal é descrever as características das tecnologias submetidas à ANS cujos RACs foram elaborados pelo Nats-HCPA no período de janeiro de 2023 a setembro de 2024.

Além disso, descrever, para cada tecnologia avaliada, a confiança nas estimativas dos tamanhos de efeitos das tecnologias avaliadas por meio do sistema GRADE (do inglês, Grading of Recommendations, Assessment, Development and Evaluation), o valor da razão de custo-efetividade incremental (RCEI) ou da razão de custo-utilidade incremental (RCUI) para o caso-base da avaliação econômica, e o valor do impacto orçamentário (IO) incremental acumulado em cinco anos.

**Método:**

Em um estudo transversal, foram analisados os dados dos Relatórios de Análise Crítica (RAC) elaborados pelo Nats-HCPA no período de dezembro de 2022 até o momento atual. Foram descritos tipo de tecnologia, tipo de doença, nível de certeza do corpo da evidência para o desfecho principal, direção do efeito, razão de custo-efetividade ou razão de custo-utilidade e a avaliação de impacto orçamentário incremental em cinco anos e parecer final. Os dados são apresentados por meio de estatística descritiva.

**Resultados:**

Foram elaborados 12 RACs, referentes a um total de dez tecnologias, sendo a indicação terapêutica em nove casos e diagnóstica em um caso. Até o momento, das 12 submissões, oito resultaram em incorporação, todas elas para tratamento. Entre as tecnologias incorporadas, quatro (50%) tiveram certeza da evidência moderada a alta, com direção de efeito para benefício. As duas tecnologias que não foram incorporadas tiveram certeza na evidência baixa ou muito baixa, uma em direção a dano e outra para benefício.

Das tecnologias avaliadas, seis (50%) não tiveram RCEI ou RCUI calculado. Das tecnologias em que o RCEI ou RCUI foi calculado, a média foi de R$ 181.256,25, sendo o maior valor R$ 419.640,00 e o menor valor R$ 646,29.

A média do AIO incremental em cinco anos foi R$ 297.571.352,91, sendo o maior valor R$ 1.301.368.772,22 e o menor valor R$ 42.378.399,99.

**Conclusão:**

A maioria das submissões resultou em incorporação da tecnologia. Tecnologias terapêuticas na área de oncologia, seguida por doenças crônicas, foram as mais frequentes. Em metade dos casos em que ocorreu incorporação, a certeza na evidência foi moderada a alta para o efeito sobre o desfecho principal, apontando para benefício. Em relação à avaliação econômica, observa-se que as duas tecnologias que não foram incorporadas apresentavam valores de RCEI e IO menores do que outras tecnologias que foram incorporadas. Isso sugere que a avaliação da eficácia e da segurança apresenta maior peso do que a avaliação econômica na decisão de incorporação.

